# Differential effects of gender on mismatch negativity to violations of simple and pattern acoustic regularities

**DOI:** 10.1002/brb3.2248

**Published:** 2021-06-14

**Authors:** Reyhane Toufan, Maryam Aghamolaei, Hasan Ashayeri

**Affiliations:** ^1^ Department of Audiology Faculty of Rehabilitation Sciences Iran University of Medical Sciences (IUMS) Tehran Iran; ^2^ Department of Audiology School of Rehabilitation Shahid Beheshti University of Medical Sciences Tehran Iran; ^3^ Department of Basic Sciences in Rehabilitation School of Rehabilitation Sciences Iran University of Medical Sciences (IUMS) Tehran Iran

**Keywords:** abstract regularities, gender, mismatch negativity, topography

## Abstract

**Introduction:**

The effects of gender on the mismatch negativity (MMN) potential have been studied using simple frequency deviants. However, the effects of gender on MMN to violations of abstract regularities have not yet been studied. Here, we addressed this issue and compared the effects of gender on simple and pattern frequency MMNs.

**Methods:**

MMN response was recorded from 29 healthy young adults, 14 females (mean age = 26.20 ± 2.17) and 15 males (mean age = 27.57 ± 2.24), using 32 scalp electrodes during simple and pattern frequency oddball paradigms and the mean amplitude, peak latency, and scalp topography of MMN evoked by each paradigm were compared between the two genders.

**Results:**

The peak latency of simple MMN was significantly longer in females (*p *< .05); however, its mean amplitude and topography were similar between the two genders (*p *> .05). There were no significant differences in peak latency, mean amplitude, and scalp topography of pattern MMN between the two genders (*p *> .05).

**Conclusions:**

Based on the obtained results, gender differently affects simple and pattern MMN. These findings may provide preliminary evidence for distinct effects of gender on various types of MMN.

## Graphical abstract

In the first report of gender effects on MMN response to violations of simple and pattern frequency regularities, we found differential effects of gender on MMN evoked by these two types of deviants; while gender had some effects on MMN evoked by simple frequency deviants, the MMN elicited by violation of pattern regularities was similar between genders. These finding provide preliminary evidence for distinct effects of gender on various types of MMN.

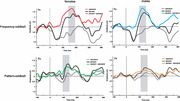

## 1 INTRODUCTION

Mismatch negativity (MMN) is a preattentive component of auditory event‐related potentials (ERPs), which was first described by Näätänen et al. in 1978 (Näätänen et al., 1978). This response is conventionally generated by presentation of an infrequent auditory stimulus (i.e., deviant) among a sequence of repetitive stimuli (i.e., standards) and its generation is mediated by two different neurophysiological mechanisms: (1) the formation of memory trace of standard stimuli and (2) change detection mechanisms that compare incoming sounds with the memory trace of previous stimuli (Bartha‐Doering et al., 2015; Yu et al., 2015). Several studies, using more complex stimulus designs, have demonstrated that memory underlying MMN generation contains sophisticated sound encoding mechanisms that can extract any regular aspect of recent stimuli, even the abstract ones hidden in the ever‐changing acoustic input (Herholz et al., 2009; Paavilainen, 2013). On the basis of these studies, change detection mechanisms detect any new event that violates the ongoing sound regularities (Garrido et al., 2009). Given the complex calculations needed for extracting abstract regularities, MMN is regarded as an index of primitive sensory intelligence in the auditory cortex (Näätänen et al., 2001, 2010).

MMN is among cognitive responses, which has found wide clinical utility (Duncan et al., 2009), providing a neurophysiological marker of auditory perceptual accuracy, general brain degeneration, and the gross functional state of the brain. Many studies have shown the applications of MMN in prediction of the functional state of patients with schizophrenia and coma outcome as well as diagnosis and treatment of impaired neurophysiological mechanisms in central auditory processing disorders, dyslexia, and Alzheimer's disease (Duncan et al., 2009; Gao et al., 2018; Kärgel et al., 2014 ; Näätänen, 2003; Näätänen & Escera, 2000; Näätänen et al., 2007, 2014; Roberts et al., 2011).

A group of studies has demonstrated that elicitation of MMN with complex stimulus designs may improve its sensitivity and specificity and also can provide new insights into the pathophysiology and management of disorders (Näätänen et al., 2014; Paavilainen, 2013; Umbricht & Krljes, 2005). Pattern regularities, regular changes of acoustic cues between several stimuli, are among complex stimulus designs that can be easily incorporated for MMN elicitation in clinical settings (Paavilainen, 2013). Pattern MMN demonstrates the capacity of the brain for extracting abstract rules, which in turn underlies our adaptive behavior in demanding and complex environments (Bendixen & Schröger, 2008; Schröger et al., 2007). Furthermore, given the importance of pitch pattern processing in the perception of speech prosody and development of word segmentation, phonological skills, and literacy, several studies have provided evidence of decreased MMN amplitude to violations of pitch patterns in patients with dyslexia and schizophrenia (Gjini et al., 2010).

For incorporating MMN evoked by complex stimulus designs in clinical settings, it is obvious that all factors potentially affecting the response should be determined in the normal population. Gender is among the important subject‐related factors that may affect the amplitude and latency of auditory evoked potentials (Hall, 2007). However, to the best of our knowledge, no study has yet investigated the effects of gender on MMN to violations of complex regularities such as pitch patterns.

Processing of pitch pattern regularities entails the sound order encoding (Alain et al., 1998), which has been reported to be stronger in males than females in some behavioral studies (Fink et al., 2005; Szymaszek et al., 2006) and similar between the two genders in others (Shrivastav et al., 2008; van Kesteren & Wiersinga‐Post, 2007). Therefore, it is important to explore whether these gender differences in auditory temporal ordering would also affect the preattentive processing of sound patterns reflected by MMN underlying mechanisms. In the present study, we investigated the effects of gender on pattern MMN evoked by the violation of a fixed pitch relation between three tones. Furthermore, given the inconsistency of scarce studies, which have addressed the gender effects on conventional MMN (Aaltonen et al., 1994; Kasai et al., 2002; Nagy et al., 2003), we also re‐examined the effects of gender on the MMN evoked by simple frequency deviants. Recording these two types of MMN in the same sample of females and males provided a unique opportunity for exploring any possible gender effect differences between various types of MMN.

## 2 METHODS

### 2.1 Participants

The participants consisted of 14 females and 15 males ranging from 23 to 30 years old with the mean ages of 26.20 ± 2.17 and 27.57 ± 2.24 years, respectively. All the subjects were monolingual and right‐handed, as measured by the Edinburg Handedness Questionnaire (EHQ), with hearing thresholds better than 20 dB HL within the frequency range of 250–8000 Hz in both ears, and with no history of otological, neurological, or psychiatric disorders and drug or alcohol abuse. Written informed consent was obtained from all participants. The research was approved by the ethics committee of Shahid Beheshti University of Medical Sciences, Tehran (Iran), and conducted according to the principles of the Declaration of Helsinki of the World Medical Association.

### 2.2 Stimuli and procedure

Participants were seated comfortably in an electrically and acoustically shielded room. A silent movie with subtitles was played on a front monitor and the subjects were instructed to watch the movie and to ignore the auditory stimuli during the experiment.

The experiment consisted of two oddball conditions, namely frequency oddball and pattern oddball. In the frequency oddball condition, the frequencies of standard and deviant stimuli were 1131 and 1269 Hz, respectively. In the pattern oddball condition, a repetitive rising pattern of pitch change between three consecutive tones (1131, 1198, and 1269 Hz) was used as the standard pattern and the reversed falling‐pitch (i.e., 1269, 1198, and 1131 Hz) was used as the deviant pattern. The auditory stimuli consisted of tone‐bursts of 50 ms duration and 5 ms rise/fall time presented binaurally using ER‐3A (Etymotic Research, Inc., Elk Grove Village, IL) insert earphones at 74 dB SPL with a stimulus‐onset asynchrony (SOA) of 180 ms. The stimuli were presented in blocks of 5 min long, and participants were given the possibility for a short break after each block. A total of 200 deviant stimuli were presented in each condition with the probability of .16.

### 2.3 Electroencephalography (EEG) recording

The EEG was recorded using a 32‐electrode cap (Wavegaurd, ANT, The Netherlands). Positions of the recording electrodes were FP1, FPz, FP2, Fz, F3, F4, F7, F8, FC1, FC2, FC5, FC6, Cz, C3, C4, T7, T8, CP1, CP2, CP5, CP6, Pz, P3, P4, P7, P8, POz, Oz, O1, O2, A1, and A2 according to the international 10–20 system. An electrode on the tip of the nose was used as the reference for all of the electrodes. The eye movements were monitored using two bipolar electrodes above and below the right eye and two electrodes at the outer canthi of each eye (vertical and horizontal electrooculograms). The impedance for all of the electrodes was maintained below 5 kΩ. The EEG signals were amplified using an ANT amplifier (Advanced Neuro Technology, Enschede, The Netherlands). The amplified signals were online band‐pass filtered from 0.05 to 500 Hz and digitized with a sampling rate of 2048 Hz using ASA 4.7.1 software (ANT, Enschede, The Netherlands).

### 2.4 EEG analysis

EEG signals were offline filtered by a band‐pass filter from 1 to 30 Hz. All electrodes were re‐referenced to the linked mastoids. Eye blinks, movement, and myogenic artifacts were removed by applying independent component analysis (Delorme & Makeig, 2004; Delorme et al., 2007). EEG data were segmented into epochs of 500 ms, including 100 ms pre‐stimulus baseline time, and were averaged separately for deviant and standard stimuli. Trials with amplitude variations exceeding ±70 μv were automatically rejected. The frequency‐change MMN response was obtained by subtracting the response of standard stimuli from that of deviant stimuli in frequency oddball condition. The pattern MMN was calculated by subtracting the response of the first tone in the standard pattern from that of the first tone in the deviant pattern (Sussman et al., 1999). MMN peak latencies were measured individually from the most negative peak at Fz at 100–250 ms poststimulus for each condition.

Individual MMN mean amplitudes were calculated from a 50‐ms time window around the individual peak latencies obtained at Fz electrode for each condition.

### 2.5 Statistical analysis

For each oddball condition, a separate repeated‐measure analysis of variance (ANOVA) including the factors stimulus type (deviant, standard) and gender (female, male) was calculated on the mean amplitudes in the MMN time range. To compare the effects of gender on simple and pattern MMNs, two separate repeated‐measure ANOVAs were performed on MMN mean amplitudes and peak latencies using the within subject factor condition (frequency oddball, pattern oddball) and the between subject factor gender (female, male). Post hoc comparisons were made using paired and independent sample *t*‐tests. Differences were considered significant when *p* < .05. Statistical package for the social sciences version 16 (SPSS Inc., IL) was used for data analysis.

Global dissimilarity index (DISS) was computed in order to compare MMN scalp topographies between the two genders without considering signal strength differences (Murray et al., 2008). DISS value measurement incorporates global field power (GFP)‐normalized voltage values of electrodes from same locations to calculate the square root of the mean of the squared voltage differences. DISS value may get any amount from zero (i.e., the compared topographic maps are completely similar) to two (i.e., the compared topographic maps are completely reversed). To determine the statistical significance of DISS values, a nonparametric randomization test was run by doing ten thousand permutations (i.e., random reassignment of each individual MMN topographic map to each gender) and calculating the grand‐average ERPs as well as DISS values of empirical topographic maps.

## 3 RESULTS

**FIGURE 1:**
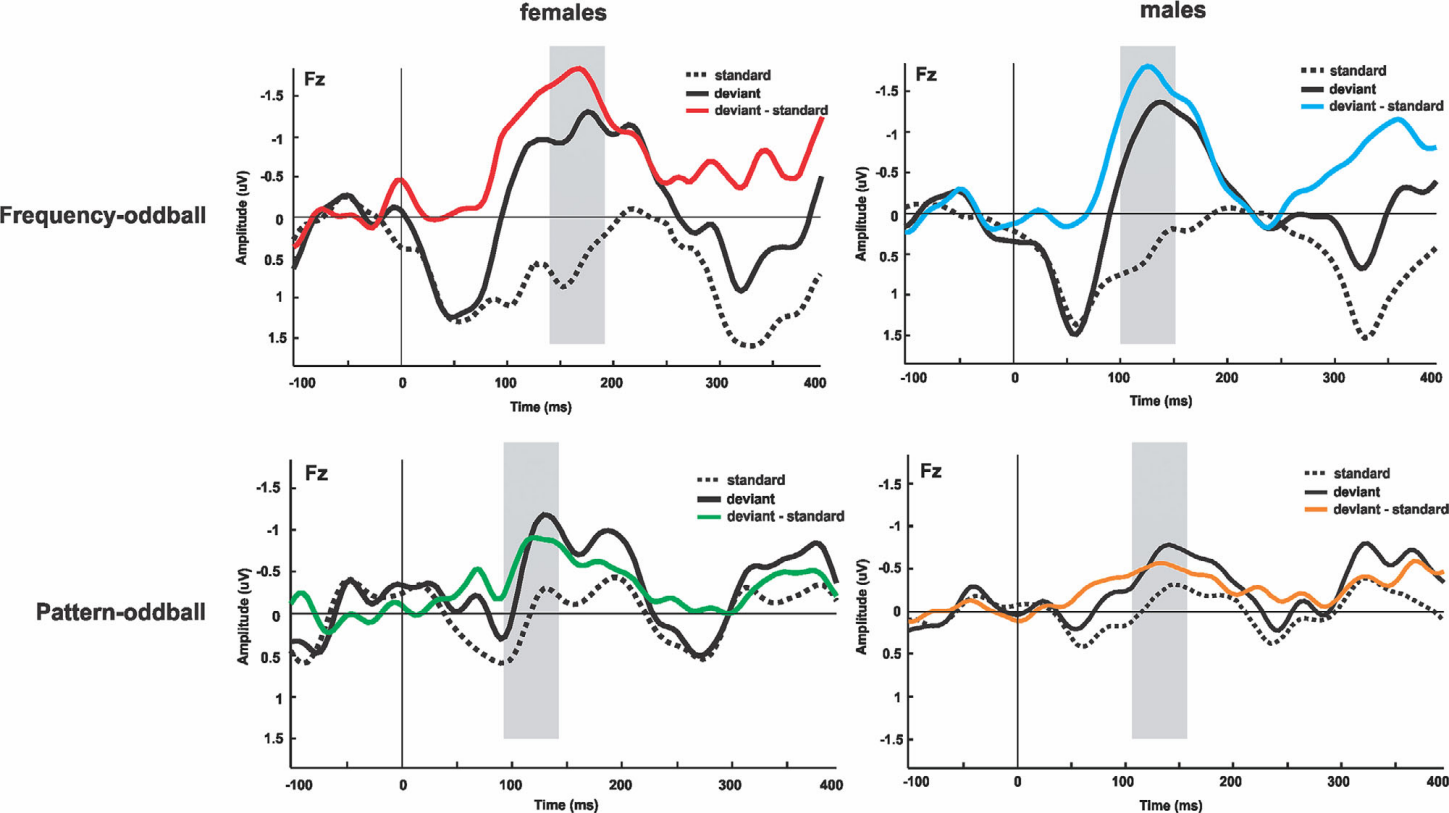
The event‐related potential (ERP) waveforms at Fz electrode. The standard, deviant, and mismatch negativity (MMN) waveforms elicited in females and males using frequency‐oddball (top) and pattern‐oddball (bottom) paradigms. MMN peak latencies of females were significantly longer than males in frequency oddball condition. The gray‐shaded bars show the time window of the mean amplitudes used for statistics

The grand‐average responses of the standard and deviant stimuli and a clear difference waveform MMN were obtained in both frequency and pattern oddball conditions (Figure 1). The mean amplitudes and peak latencies for simple frequency‐change and pattern MMN responses are given in Table 1. In both oddball conditions, repeated‐measure ANOVA revealed a main effect of stimulus type (deviant, standard) on mean amplitudes in the time window of MMN (frequency oddball condition: *F*_1,27 _= 98.31, *p *< .001, $\eta {\;}_{P}^{2}$ = 0.78; pattern oddball condition: *F*_1,27 _= 51.43, *p *< .001, $\eta {\;}_{P}^{2}$= 0.65) in terms of larger amplitudes elicited by deviant compared to standard stimuli, but no stimulus type X gender interaction was found for the mean amplitudes in any conditions (frequency oddball condition: *F*_1,27_ = 1.39, *p* = .24, $\eta {\;}_{P}^{2}$ = 0.04; pattern oddball condition: *F*_1,27_ = 0.83, *p* = .37, $\eta {\;}_{P}^{2}$ = 0.03).

**TABLE 1:** The averages of mean amplitudes and peak latencies of mismatch negativity (MMN) responses for the two experimental conditions in females and males

	Frequency‐change MMN		Pattern MMN	
	Females (*n* = 14)	Males (*n* = 15)	Females (*n* = 14)	Males (*n* = 15)
Mean amplitude (μv)	1.89 (0.25)	1.48 (0.23)	1.03 (0.10)	0.79 (0.22)
Peak latency (ms)	173.55 (9.06)	142.58 (6.76)	150.11 (8.32)	158.08 (7.49)

Standard errors of mean (SEM) are given in parentheses.

**FIGURE 2:**
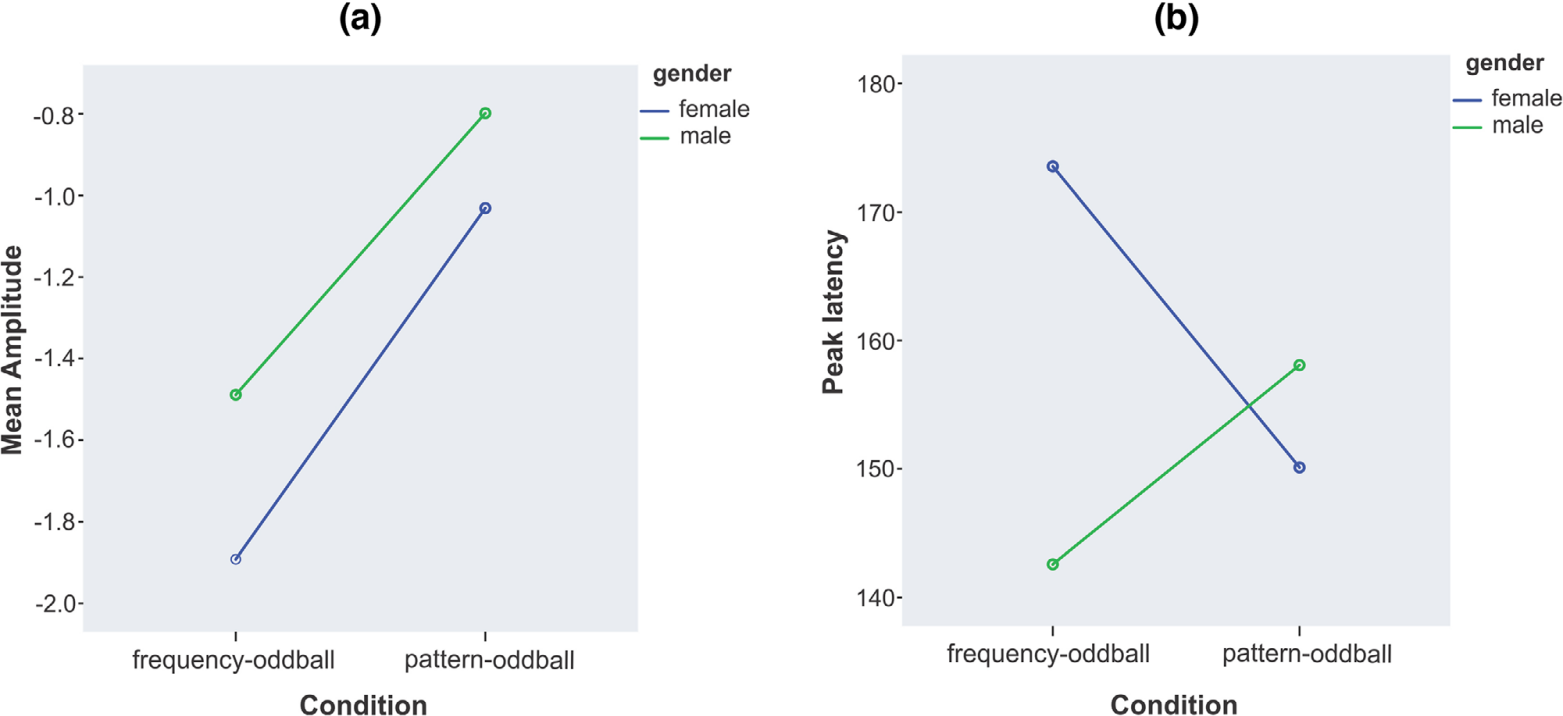
Repeated‐measures analyses of variance (ANOVAs): Gender × condition interaction graphs for (a) mismatch negativity (MMN) mean amplitudes (b) MMN peak latencies. The graph illustrates that gender can modulate the effects of oddball conditions (simple, pattern) on MMN peak latencies but has no effects on mean amplitudes

Repeated‐measure ANOVA on MMN mean amplitudes revealed a main effect of condition (*F* = 15.68, *p *< .001, $\eta {\;}_{P}^{2}$ = 0.36) in terms of larger MMN amplitudes for frequency versus pattern oddball condition. However, no gender × condition interaction was found for MMN mean amplitudes (*F*_1,27_ = 0.19, *p* = .66, $\eta {\;}_{P}^{2}$ = 0.007) (Figure 2a).

**FIGURE 3:**
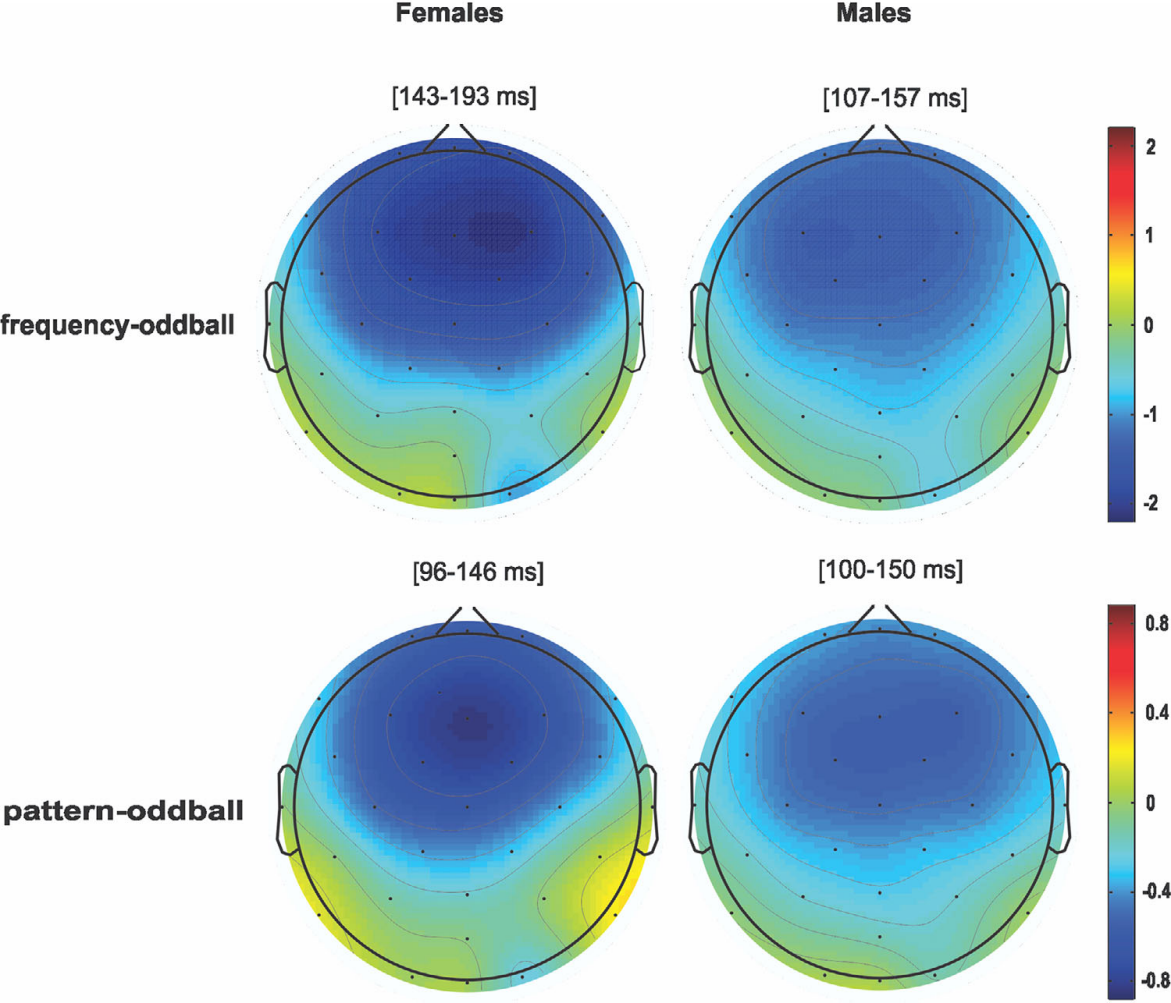
Topographic distributions of mismatch negativity (MMN) response. MMN topographic maps of females and males for frequency‐oddball (top) and pattern‐oddball (bottom) conditions

Repeated‐measure ANOVA on MMN peak latencies showed no main effect of condition (*F*_1,27_ = 0.30, *p* = .58, $\eta {\;}_{P}^{2}$ = 0.01) but a significant gender × condition interaction (*F*_1,27_ = 7.22, *p* = .01, $\eta {\;}_{P}^{2}$ = 0.21) was observed for MMN peak latencies (Figure 2b). Post hoc comparisons revealed that in females, MMN peak latencies were significantly longer for frequency compared to pattern oddball condition (*t*_(13)_ = 2.67, *p* = .01, *d* = 0.65) but no significant difference was found between the peak latencies of frequency and pattern MMN in males (*t*_(14)_ = −1.36, *p* = .19, *d* = 0.36). Furthermore, MMN peak latencies of females were significantly longer than males in frequency oddball condition (*t*_(27)_ = 2.76, *p* = .01, *d* = 1.02); however, no significant difference was found between MMN peak latencies of females and males in pattern oddball condition (*t*_(27)_ = −0.71, *p* = .48, *d* = 0.26). Figure 3 demonstrates the topographic distributions of simple and pattern MMNs in females and males. There was no significant difference in topographic distribution of simple and pattern MMNs between the two genders (simple: DISS = 0.27, *p* = .43; pattern: DISS = 0.40, *p* = .63).

## 4 DISCUSSION

The present study examined the effects of gender on two types of MMN evoked by simple frequency changes and pitch pattern violations. In the frequency oddball condition, MMN peak latency was significantly increased in females compared to males, whereas its mean amplitude and topographic distribution was similar between the two genders. In the pattern oddball condition, no effect of gender on the mean amplitude, peak latency, and topographic map of MMN was found.

The similarity of pattern MMN between females and males obtained in the present study shows that automatic encoding of pattern regularities and detecting the corresponding deviations is not affected by gender. Some previous behavioral studies have demonstrated the effect of gender on temporal ordering tasks (Fink et al., 2005; Szymaszek et al., 2006). Given that the neural representation of pitch patterns is achieved by encoding the temporal order of pattern‐composing stimuli (Alain et al., 1998), one might expect a corresponding effect of gender on pattern MMN. However, our findings can indicate that the differences between females and males in temporal ordering behavioral tasks are not likely reflected in the pre‐attentive processing of pitch patterns within the MMN time range. These results preclude the need for gender specific normative data for clinical interpretation of pattern MMN in the adult population.

In the present study, the mean amplitude of MMN to frequency changes was similar between the two genders; however, its peak latency was longer in females. Nagy et al. (2003) and Tsolaki et al. (2015) also investigated the effects of gender on MMN evoked by frequency changes and found no differences in MMN mean amplitudes or peak latencies between females and males. Different effect of gender on MMN peak latency observed in the present study compared to the above‐mentioned studies might origin from the small magnitude of change (two semitones) used in the current study. Previous studies have shown that neural generators of MMN evoked by small and large frequency changes are different and there is more in common between neural generators of N1 and MMN for large changes compared to small ones (Nagy et al., 2003; Opitz et al., 2002). So, opposed to Nagy et al. (2003) and Tsolaki et al. (2015) studies that probably reflected the mixed effects of gender on both MMN and N1 responses, due to large magnitude of changes used in their stimulus designs, findings of the present study might represent the mere effects of gender on MMN generators.

Different effects of gender on simple and pattern MMN observed in the current study indicate that MMN elicited by various types of deviants might be differentially affected by gender. This interpretation gets further support from Matsubayashi et al. (2008) and Fan et al. (2013) studies, which recorded MMN responses in the same sample of subjects using different stimulus paradigms. Matsuayashi et al. (2008) found longer latencies in females compared to males for phoneme MMNm but no difference between the two genders in terms of MMNm amplitude or latency using frequency and duration deviants. Fan et al. (2013) also reported a significant effect of sex on MMN to emotionally rich syllables but not to emotionless nonvocal sounds with similar acoustic content. The notion of different effects of gender on different types of MMN can also shed light on the discrepancy of present findings with previous ones that used different types of stimuli for MMN elicitation (Barrett & Fulfs, 1998; Kasai et al., 2002; Matsubayashi et al., 2008 ). For example, Aaltonen et al. (1994) found longer latencies for phonetic MMN in females than in males but Kasai et al. (2002) showed no difference in MMN amplitude and peak latency between the two genders using duration deviants. On the other hand, Schirmer et al. (2005) and Barrett and Fulfs (1998) reported larger MMN amplitudes in females compared to males with no latency differences between two sexes using emotional voice and intensity deviants, respectively. These inconsistent findings might be explained by the fact that various types of deviants activate separate MMN neural networks (Escera et al., 2002; Molholm et al., 2005; Toufan et al., 2016), which can be affected differently by gender.

Altogether, our results showed differential effects of gender on MMN evoked by the violation of simple versus pattern acoustic regularities. The implications of these findings could be helpful for the interpretation of MMN results in healthy and/or clinical populations. Furthermore, our findings provide preliminary evidence for the claim that gender might distinctly affect various types of MMN. More research is needed to further explore MMN gender differences by various stimulus designs.

## CONFLICT OF INTEREST

The authors declare no conflict of interest.

### PEER REVIEW

The peer review history for this article is available at https://publons.com/publon/10.1002/brb3.2248.

## Data Availability

The data that support the findings of this study are available from the corresponding author upon reasonable request.
